# Association between metabolic score for insulin resistance and clinical outcomes: insights from the Tehran lipid and glucose study

**DOI:** 10.1186/s12986-024-00808-w

**Published:** 2024-06-12

**Authors:** Seyyed Saeed Tamehri Zadeh, Neda Cheraghloo, Soroush Masrouri, Farzad Esmaeili, Fereidoun Azizi, Farzad Hadaegh

**Affiliations:** 1 Prevention of Metabolic Disorders Research Center, Research Institute for Endocrine Sciences, Shahid Beheshti University of Medical Sciences, No. 24, Yamen Street, P.O. Box: 19395-4763, Velenjak, Tehran, Iran; 2 Department of Epidemiology and Biostatistics, School of Public Health, Tehran University of Medical Sciences, Tehran, Iran; 3 Endocrine Research Center, Research Institute for Endocrine Sciences, Shahid Beheshti University of Medical Sciences, Tehran, Iran

**Keywords:** Metabolic score for insulin resistance, Coronary heart disease, Stroke, Mortality, Diabetes, Hypertension, Chronic kidney disease

## Abstract

**Background:**

We aimed to assess the relationship between Metabolic Score for Insulin Resistance (METS-IR) and the incidence of coronary heart disease (CHD), stroke, mortality, diabetes, hypertension, and chronic kidney disease (CKD) in a population from the Middle East and North Africa (MENA) region.

**Method:**

Individuals aged ≥ 20 years were enrolled. Cox proportional hazards regression models were applied to assess the association between METS-IR and incident CHD, stroke, all-cause mortality, diabetes, hypertension, and CKD.

**Results:**

Over a median follow-up period of 9–18 years, 1080 (10.6%), 267 (2.6%), 1022 (9.6%), 1382 (16.4%), 2994 (58.5%), and 2002 (23.0%) CHD, stroke, all-cause mortality, diabetes, hypertension, and CKD events occurred, respectively. Compared to the lowest quartile (reference), the hazard ratios (HR) associated with the highest quartile of METS-IR were 1.527 (95% confidence interval [CI]: 1.208–1.930, *P* for trend 0.001), 1.393 (0.865–2.243, > 0.05), 0.841 (0.682–1.038, > 0.05), 3.277 (2.645–4.060, < 0.001), 1.969 (1.752–2.214, < 0.001), and 1.020 (0.874–1.191, > 0.05) for CHD, stroke, all-cause mortality, diabetes, hypertension, and CKD, respectively. METS-IR, as a continuous variable, was significantly associated with the risk of incident CHD [HR, 95% CI: 1.106, 1.034–1.184], diabetes [1.524, 1.438–1.616], and hypertension [1.321, 1.265–1.380]. These associations were also independent of metabolic syndrome (METS) and remained unchanged in a subgroup of individuals without METS and/or diabetes.

**Conclusions:**

Increasing levels of METS-IR were significantly associated with a greater risk of incident CHD, diabetes, and hypertension; therefore, this index can be a useful tool for capturing the risk of these clinical outcomes.

## Introduction

Insulin resistance (IR) is a key player in the development of cardiometabolic disorders, including diabetes, hypertension, chronic kidney disease (CKD), and cardiovascular disease (CVD), the subsequent rise of which has made them a major cause of mortality and morbidity globally [1]; notably, the Middle East and North Africa (MENA) region has the highest age-standardized total diabetes prevalence rates, at the super-region level [2].

The MENA region faces a substantial burden of non-communicable diseases (NCDs), with a notable prevalence of obesity, hypertension, and IR; the MENA region, marked by diverse social development levels, has seen significant shifts in its social, economic, and cultural fabric. Risk factors contributing to this prevalence, such as tobacco use, unhealthy diets, and physical inactivity, are widespread [3, 4]. Alarming obesity rates, affecting both adults and children, compound the issue, with 17% of deaths and 11% of disability-adjusted life years (DALYs) attributed to excess body weight in the region [5, 6]. Research within the Iranian population has estimated that 33.8% of the prevalence of diabetes can be attributed to obesity [7]. Energy-dense diets, heavy on saturated fats and refined carbohydrates while lacking in fruits and vegetables, exacerbate this trend [8]. Sedentary lifestyles further amplify the risk, surpassing global averages [9].

Despite these challenges, policy responses to NCDs remain inadequate [3, 10], and the projection of metabolic health for the future is concerning, with trends indicating a continued rise in NCDs [10, 11]. This alarming trend underscores the urgent need to understand the full spectrum of IR’s impact on health outcomes.

The Metabolic Score for Insulin Resistance (METS-IR), presented by Bello-Chavolla et al. in 2018, offers a non-insulin-based alternative to traditional methods for quantifying peripheral insulin sensitivity, using easily obtainable fasting laboratory values, including fasting plasma glucose (FPG), triglycerides (TG), high-density lipoprotein cholesterol (HDL-C), and body mass index (BMI) [12]. Designed to overcome the limitations of more complex and invasive techniques like the euglycemic hyper-insulinemic clamp, METS-IR has shown a high predictive value.

It better predicts coronary artery calcium (CAC) score compared to TG to HDL-C ratio (TG/HDL-C) [13], diabetes compared to triglyceride-glucose (TyG) index and TG/HDL-C [12], and major adverse cardiac events compared to homeostatic model assessment of IR (HOMA-IR), TyG, TyG-BMI, TyG-waist-to-height ratio (TyG-WHTR), and TyG-waist circumference (TyG-WC) [14]. Its significance lies in its ability to effectively assess IR, a key factor in the development of metabolic syndrome (METS) and CVD, thereby aiding in predicting and managing these conditions [15]. However, its performance across various ethnicities requires further study to fully validate its universal applicability [16].

METS-IR is known to be associated with various adverse clinical outcomes, including coronary heart disease (CHD) [17], stroke [18], diabetes [12], hypertension [19, 20], and CKD [21]. However, there was no correlation between METS-IR and coronary artery diseases in a case-control study among Iranian patients in the fully adjusted model [22]. Previous research has established connections between IR and these health outcomes [23, 24], but comprehensive studies examining these relationships in the context of METS-IR are lacking.

We aimed to investigate the associations of METS-IR with various health outcomes, including CHD, stroke, all-cause mortality, diabetes, hypertension, and CKD, in the context of the Tehran Lipid and Glucose Study (TLGS), the oldest cohort in the MENA region. Additionally, we examined whether these relationships exist in individuals without METS and/or diabetes.

## Methods

### Study design and setting

The TLGS is a community-based prospective cohort study initially designed to investigate the risk factors for NCDs in a representative population of Tehran, Iran. Participants aged ≥ 3 years were recruited in two phases, including phase I (1999–2001) and II (2002–2005), bringing the total cohort study population to 18,555 individuals; data collection continued in about 3-year intervals in the follow-up phases (phases III, IV, V, and VI). Details of the design have been published elsewhere [25]. For this study, 12,790 participants aged ≥ 20 years (10,362 enrolled in phase I and 2,428 enrolled in phase II were selected.

### Study population

**Fig. 1:**
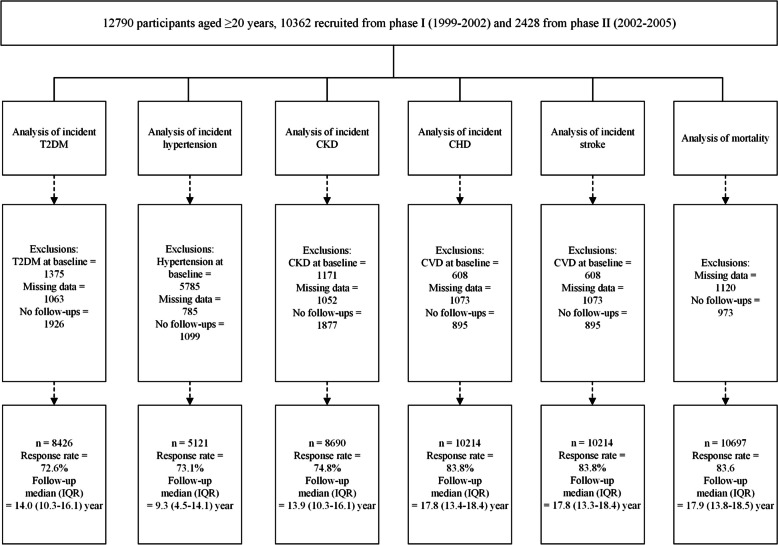
Flowchart of the study population, Tehran Lipid and Glucose Study, 1999–2018. T2DM, type 2 diabetes; CKD, chronic kidney disease; CHD, coronary heart disease; CVD, cardiovascular disease; IQR, interquartile range

Figure 1 demonstrates the details of the study population regarding the exclusion criteria for each outcome, response rates, and outcome-specific follow-up duration. For the analysis of each outcome, certain exclusion criteria were carried out; accordingly, regarding diabetes, after excluding those with baseline diabetes (*n* = 1375), or missing covariates used in diabetes models (*n* = 1063), or no available follow-up data (*n* = 1926), 8426 participants remained. For hypertension analysis, after the exclusion of those with prevalent hypertension (*n* = 5785), missing covariates (*n* = 785), or no available follow-up data (*n* = 1099), 5121 participants remained. Regarding CKD, after excluding those with prevalent CKD (*n* = 1171), missing covariates (*n* = 1052), or no follow-up (*n* = 1877), 8690 participants remained for the analysis. For stroke and CHD analyses, after excluding individuals with prevalent CVD (*n* = 608), missing covariates (*n* = 1073), or no available follow-up data (*n* = 895), 10,214 participants remained. Eventually, for mortality, individuals with missing covariates (*n* = 1120) or without any follow-up data (*n* = 973) were excluded, leaving 10,697 participants for the analysis. Participants were followed up until March 2018. Participant response rates ranged from 72.6% (for diabetes) to 83.8% (for CHD/stroke events).

Written informed consent was obtained from all of the individuals who participated in the study, which was approved by the Ethical Committee of the Research Institute for Endocrine Sciences, Shahid Beheshti University of Medical Sciences, Tehran, Iran.

### Clinical and laboratory measurements

Details on the laboratory procedures protocols of TLGS have been published previously [25]. Using interviewer-administered and standardized questionnaires, demographics, past medical and medication history, family history of CVD and diabetes, and smoking status data were obtained. Details on anthropometric assessment, measurement of blood pressure, and blood lipids have been explained elsewhere [25–27]. Resting heart rate was the average of 2 times measuring the radial artery pulse over 1 min. Blood samples were collected between 07:00 and 09:00 A.M., after at least 12 h of fasting, and analyzed on the same day of blood sample collection. Further details of FPG, 2-hour plasma glucose (2 h-PG), and serum creatinine have been reported elsewhere [25, 28]. The estimated glomerular filtration rate (eGFR) was estimated by the Chronic Kidney Disease Epidemiology Collaboration equation [29].

### Definition of terms

The participants were divided based on their smoking status into two categories: current smokers and past/never smokers. The presence of a family history of premature CVD was positive, with a history of CHD/stroke in a first-degree relative (before the age of 55 years for male and 65 years for female relatives). The presence of a family history of diabetes was positive with having a first-degree relative with diabetes. BMI was calculated as weight in kilograms divided by height in square meters. Prediabetes was defined as having untreated FPG of 5.6–6.9 mmol/L (100–125 mg/dL) or a 2 h-PG level during an oral glucose tolerance test (OGTT) of 7.8–11.0 mmol/L (140–199 mg/dL). Diabetes was defined with FPG ≥ 7.0 mmol/L (126 mg/dL), 2 h-PG ≥ 11.1 mmol/L (200 mg/dL), or taking glucose-lowering medication(s). Elevated blood pressure was defined with untreated systolic blood pressure (SBP) of 120–129 mmHg and diastolic blood pressure (DBP) < 80 mmHg. Hypertension was defined as having SBP ≥ 130 mmHg, DBP ≥ 80 mmHg, or taking anti-hypertensive medication(s). Furthermore, CKD was defined with an eGFR < 60 mL/min per 1.73 m^2^. METS-IR was calculated as ln (2 × FPG [mg/dL] + TG [mg/dL]) × BMI (kg/m^2^)/ln (HDL-C [mg/dL]) [12]. METS was defined according to the JIS (Joint Interim Statement), including SBP, DBP, FPG, TG, HDL-C levels, and abdominal obesity defined with the appropriate population-specific cutoff points for waist circumference (95 cm for men and women) [30–32].

A skilled physician collected complementary information about each medical event leading to hospitalization either during home visits or from sources, including hospital records, death certificates, forensic medical reports, or verbal autopsies when necessary [25, 33]. The diagnosis of stroke and CHD was according to the criteria of the International Classification of Diseases (ICDs), 10th Revision (CHD: Rubric I20–I25; stroke: Rubric I60–I69, and G45). The Cohort Outcome Committee, composed of an internist, an endocrinologist, a cardiologist, the physician who collected the information, and other invited specialists, when necessary, adjudicated the outcomes.

### Statistical analysis

We presented the continuous variables with mean (standard deviation) and categorical variables with frequencies (percentage). When skewed, we presented a variable as the median (interquartile range). The baseline characteristics of the study participants were compared according to quartiles of METS-IR using the ANOVA test for continuous variables and the Chi-squared test for categorical variables; for skewed variables, the Kruskal-Wallis test was used for comparison.

A multivariable-adjusted restricted cubic splines analysis was conducted in Cox regression hazard models to explore the shape of the association between METS-IR and different outcomes with 4 knots (5th, 35th, 65th, and 95th percentiles of METS-IR index). Cox proportional hazard regression models were used to assess the association of METS-IR (both per 1-SD and in quartiles) with the incidence of CHD, stroke, all-cause mortality, diabetes, hypertension, and CKD. These associations were assessed in age- and gender-adjusted models (Model 1) and multivariable-adjusted models (Model 2); covariates in Model 2 included age, gender, current smoking, diabetes (prediabetes for the outcome of diabetes), hypertension (elevated blood pressure for the outcome of hypertension), prevalent CVD (family history of premature CVD for the outcome of CHD and stroke), family history of diabetes (only for the diabetes models), non-HDL-C, lipid-lowering drug use, pulse rate, and serum creatinine. The proportionality assumption in the Cox regression models was tested, and stratified multivariable Cox regression models were fitted with age quantiles as a stratifying factor. We further adjusted for the METS in Model 3 as a secondary analysis. For another secondary analysis, we repeated the analyses among individuals without diabetes and/or METS to explore whether the same association exists in individuals without these conditions.

For the CHD, stroke, and mortality outcomes, the event date was defined with the exact date of the incidence of the event. The event date for incident diabetes, hypertension, and CKD cases was defined with the mid-time between the date of the follow-up visit at which an outcome was ascertained for the first time and the most recent follow-up visit preceding the diagnosis. We defined censoring as being lost to follow-up or reaching the end of the study. Follow-up time was calculated as the difference between the time of study entrance and either the event date (exact or calculated mid-time date, as appropriate) or censoring, whichever happened first. We performed subgroup analyses and checked for age (< 55 and ≥ 55 years), gender, diabetes (prediabetes for the outcome of diabetes), and hypertension (elevated blood pressure for the outcome of hypertension) interaction with the association of METS-IR and different outcomes. Analyses were performed with STATA version 14 SE (Stata Corp LP, TX, USA) and R version 3.6.2. A two-tailed *P* value of < 0.05 is considered statistically significant.

## Results

Baseline characteristics according to quartiles of METS-IR are shown in Table 1. Generally, compared to the lowest quartile of METS-IR, those in the highest quartile were older and had worse cardiometabolic status. Additionally, a higher proportion of women was observed in the lowest and highest quartiles compared to the other quartiles. Moreover, the prevalence of CVD, diabetes, hypertension, CKD, and METS and the use of lipid-lowering, glucose-lowering, and anti-hypertensive medications was higher in the highest quartile of METS-IR.

**Table 1:** Characteristics of the study participants according to the quartiles of METS-IR: Tehran Lipid and Glucose Study

Variables	Quartile 1 (20.6-35.7) (*n* = 2,675)	Quartile 2 (35.8-42.0) (*n* = 2,674)	Quartile 3 (42.1-48.7) (*n* = 2,674)	Quartile 4 (48.8-95.6) (*n* = 2,674)	*P*-value
**Continuous variables, Mean (SD)**					
Age, mean (SD), year	35.35 (14.79)	42.50 (15.03)	45.07 (13.80)	45.81 (12.75)	<0.001
BMI, mean (SD), kg/m^2^	21.46 (2.23)	25.32 (1.98)	28.12 (2.33)	32.12 (3.91)	<0.001
WC, mean (SD), cm	74.87 (7.32)	85.46 (7.36)	92.15 (7.65)	100.90 (9.15)	<0.001
Pulse rate, mean (SD), beats/min	79.37 (11.83)	78.61 (11.72)	78.68 (11.35)	79.74 (11.24)	0.004
FPG, mean (SD), mg/dL	87.44 (16.90)	93.10 (24.35)	99.28 (30.80)	110.49 (44.69)	<0.001
2 h-PG, mean (SD), mg/dL	96.40 (33.85)	109.46 (42.23)	120.46 (52.81)	139.08 (71.61)	<0.001
SBP, mean (SD), mmHg	110.14 (15.49)	117.73 (18.18)	121.89 (19.29)	125.76 (19.45)	<0.001
DBP, mean (SD), mmHg	71.67 (9.82)	76.09 (10.31)	78.79 (10.47)	81.97 (10.63)	<0.001
Serum creatinine, mean (SD), mg/dL	1.02 (0.17)	1.07 (0.17)	1.08 (0.23)	1.07 (0.17)	<0.001
TC, mean (SD), mg/dL	180.48 (38.96)	203.47 (43.22)	214.17 (45.14)	220.98 (48.27)	<0.001
Non-HDL-C, mean (SD), mg/dL	131.85 (37.41)	160.31 (41.15)	174.13 (43.19)	185.45 (46.57)	<0.001
HDL-C, mean (SD), mg/dL	48.43 (11.20)	42.95 (9.72)	39.91 (9.40)	35.53 (8.99)	<0.001
TG, median (IQR), mg/dL	85 (65-111)	126 (94-170)	164 (123-223)	218 (162-302)	0.001
**Categorical variables, number (%)**					
Women	1544 (57.72)	1439 (53.81)	1439 (53.81)	1567 (58.60)	<0.001
Current smoking	443 (16.56)	437 (16.34)	406 (15.18)	431 (16.12)	0.534
Family history of CVD	277 (10.36)	326 (12.19)	432 (16.16)	409 (15.30)	<0.001
Prevalent CVD	53 (1.98)	108 (4.04)	152 (5.68)	168 (6.28)	<0.001
Family history of diabetes	570 (21.31)	695 (25.99)	813 (30.40)	906 (33.88)	<0.001
Diabetes	68 (2.54)	170 (6.36)	332 (12.42)	613 (22.92)	<0.001
Glucose-lowering medication	28 (1.05)	83 (3.10)	128 (4.79)	215 (8.04)	<0.001
Hypertension	643 (24.04)	1,131 (42.30)	1,417 (52.99)	1,750 (65.45)	<0.001
Anti-hypertensive medication	61 (2.28)	162 (6.06)	256 (9.57)	315 (11.78)	<0.001
Lipid-lowering medication	18 (0.67)	59 (2.21)	93 (3.48)	168 (6.28)	<0.001
CKD	99 (3.75)	254 (9.48)	278 (10.32)	336 (12.52)	<0.001
Metabolic syndrome	62 (2.32)	471 (17.61)	1,152 (43.08)	2,091 (78.20)	<0.001

Data were given as mean (SD) or number (%), except for skewed variables (i.e., TG)

*Abbreviations*: *METS-IR* Metabolic Score for Insulin Resistance, *BMI* Body mass index, *WC* Waist circumference, *FPG* Fasting plasma glucose, *2 h-PG* 2-hour post-challenge plasma glucose, *SBP* Systolic blood pressure, *DBP* Diastolic blood pressure, *TC* Total cholesterol, *HDL-C* high-density lipoprotein cholesterol, *TG* Triglycerides, *CVD* Cardiovascular disease, *CKD* Chronic kidney disease

**Fig. 2:**
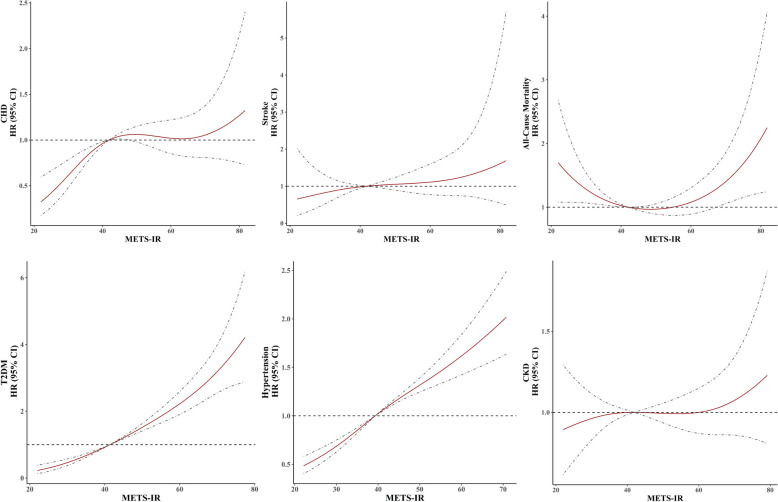
Restricted cubic splines for the relationship between METS-IR and the hazard ratios for CHD, stroke, all-cause mortality, diabetes, hypertension, and CKD. Adjusted for age, gender, current smoking, diabetes (prediabetes for the outcome of diabetes), hypertension (elevated BP for the outcome of hypertension), prevalent CVD (family history of CVD for the outcomes of stroke and CHD), family history of diabetes (for the outcome of diabetes), non-HDL-C, lipid-lowering drug use, pulse rate, and serum creatinine. *Abbreviations*: METS-IR, Metabolic Score for Insulin Resistance; CHD, coronary heart disease; T2DM, type 2 diabetes; CKD, chronic kidney disease; CVD, cardiovascular disease; BP, blood pressure; HDL-C, high-density lipoprotein cholesterol; HR, hazard ratio; CI, confidence interval

The association of METS-IR with diabetes, hypertension, CKD, CHD, stroke, and all-cause mortality is shown in Fig. 2. The association of METS-IR and all-cause mortality was U-shaped.

The subjects were followed for approximately 17.9 years for incident CHD, stroke, and all-cause mortality, 14.0 years for diabetes and CKD, as well as 9.3 years for hypertension. Over the follow-up, 1080 (10.6%), 267 (2.6%), 1022 (9.6%), 1382 (16.4%), 2994 (58.5%), and 2002 (23.0%) CHD, stroke, all-cause mortality, diabetes, hypertension, and CKD events occurred, respectively.

The association of METS-IR with CHD, stroke, and all-cause mortality is shown in Table 2. In the model adjusted for age, gender, current smoking, diabetes, hypertension, family history of premature CVD, non-HDL-C, lipid-lowering drug use, pulse rate, and serum creatinine, for the outcome of CHD, the hazard ratio (HR) associated with second, third, and the highest quartiles of METS-IR, compared to the lowest quartile, was 1.323 (95% Confidence Interval [CI]; 1.046–1.673), 1.543 (1.226–1.942), and 1.527 (1.208–1.930), respectively (*P* for trend = 0.001). Each 1-SD greater METS-IR value was associated with a 1.106 times higher risk of CHD [HR, 95% CI; 1.106, 1.034–1.184]. Regarding stroke events, compared to the lowest quartile, the HRs for the second, third, and highest quartiles were 1.356 (0.854–2.154), 1.477 (0.931–2.343), and 1.393 (0.865–2.243), respectively, in model 2 (*P* for trend = 0.28). Regarding all-cause mortality, the HRs for the second, third, and highest quartiles were 0.839 (0.689–1.022), 0.807 (0.660–0.986), and 0.841 (0.682–1.038) (*P* for trend = 0.18). In a secondary analysis, after adjustment with METS, the results remained generally unchanged (Table 2).

**Table 2:** Hazard ratios and 95% confidence intervals of incident coronary heart disease, stroke, and all-cause mortality associated with the Metabolic Score for Insulin Resistance

	Event (%)	Model 1	*P*-value	Model 2	*P*-value	Model 3	*P*-value
**Coronary heart disease**^a^							
Quartile 1	102 (3.89)	1 (Reference)		1 (Reference)		1 (Reference)	
Quartile 2	241 (9.40)	1.556 (1.233-1.964)	<0.001	1.323 (1.046-1.673)	0.019	1.291 (1.018-1.636)	0.034
Quartile 3	332 (13.17)	2.062 (1.648-2.580)	<0.001	1.543 (1.226-1.942)	<0.001	1.447 (1.137-1.842)	0.003
Quartile 4	405 (16.17)	2.552 (2.047-3.183)	<0.001	1.527 (1.208-1.930)	<0.001	1.389 (1.075-1.796)	0.012
P for trend		-	<0.001	-	0.001	-	0.034
Per SD		1.349 (1.270-1.433)	<0.001	1.106 (1.034-1.184)	0.003	1.065 (0.986-1.150)	0.108
**Stroke**^a^							
Quartile 1	26 (0.99)	1 (Reference)		1 (Reference)		1 (Reference)	
Quartile 2	67 (2.51)	1.590 (1.007-2.508)	0.046	1.356 (0.854-2.154)	0.196	1.340 (0.838-2.142)	0.221
Quartile 3	82 (3.25)	1.976 (1.264-3.089)	0.003	1.477 (0.931-2.343)	0.097	1.437 (0.878-2.352)	0.148
Quartile 4	92 (3.67)	2.289 (1.466-3.576)	<0.001	1.393 (0.865-2.243)	0.172	1.345 (0.796-2.273)	0.268
P for trend		-	<0.001	-	0.277	-	0.451
Per SD		1.309 (1.153-1.486)	<0.001	1.094 (0.948-1.262)	0.218	1.079 (0.919-1.267)	0.351
**All-cause mortality**							
Quartile 1	184 (6.88)	1 (Reference)		1 (Reference)		1 (Reference)	
Quartile 2	252 (9.42)	0.895 (0.739-1.085)	0.261	0.839 (0.689-1.022)	0.082	0.832 (0.680-1.017)	0.074
Quartile 3	289 (10.81)	1.005 (0.832-1.213)	0.954	0.807 (0.660-0.986)	0.037	0.792 (0.636-0.986)	0.037
Quartile 4	297 (11.11)	1.179 (0.974-1.428)	0.090	0.841 (0.682-1.038)	0.107	0.821 (0.646-1.043)	0.108
P for trend		-	0.019	-	0.181	-	0.183
Per SD		1.121 (1.047-1.201)	0.001	0.983 (0.910-1.061)	0.666	0.987 (0.904-1.077)	0.770

Model 1: adjusted for age and gender

Model 2: Model 1 + current smoking, diabetes, hypertension, family history of premature CVD (prevalent CVD for the outcome of mortality), non-HDL-C, lipid-lowering drug use, pulse rate, and serum creatinine

Model 3: Model 2 + metabolic syndrome

*Abbreviations*: *SD* Standard deviation, *CHD* Coronary heart disease, *CVD* Cardiovascular disease, *HDL-C* High-density lipoprotein cholesterol

^a^For the analysis of CHD and stroke, individuals with prevalent CVD are excluded

The association of METS-IR with diabetes, hypertension, and CKD is shown in Table 3. Compared to the reference quartile, adjusted HRs (95% CI) for incident diabetes for the second, third, and highest quartiles were 1.583 (1.270–1.974), 2.249 (1.817–2.786), and 3.277 (2.645–4.060), respectively; the corresponding HRs for incident hypertension were 1.286 (1.161–1.424), 1.648 (1.480–1.835), and 1.969 (1.752–2.214), respectively (both *P* for trends < 0.001). Regarding CKD, only in the gender- and age-adjusted model, significant risk associated with METS-IR was observed, with HRs of 1.195 (1.033–1.382), 1.177 (1.020–1.357), and 1.266 (1.097–1.461), respectively, for the second, third, and fourth quartiles (*P* for trend 0.005). Each 1-SD greater METS-IR was also associated with 52% [HR, 95% CI; 1.524, 1.438–1.616] and 32% [1.321, 1.265–1.380] higher diabetes and hypertension risk in model 2, respectively.

**Table 3:** Hazard ratios and 95% confidence intervals of incident diabetes, hypertension, and chronic kidney disease associated with the Metabolic Score for Insulin Resistance

	Event (%)	Model 1	*P*-value	Model 2	*P*-value	Model 3	*P*-value
**Diabetes**							
Quartile 1	120 (5.36)	1 (Reference)		1 (Reference)		1 (Reference)	
Quartile 2	272 (12.26)	1.704 (1.369-2.120)	<0.001	1.583 (1.270-1.974)	<0.001	1.557 (1.248-1.942)	<0.001
Quartile 3	418 (19.69)	2.522 (2.045-3.109)	<0.001	2.249 (1.817-2.786)	<0.001	2.114 (1.698-2.631)	<0.001
Quartile 4	572 (31.02)	3.823 (3.113-4.695)	<0.001	3.277 (2.645-4.060)	<0.001	2.927 (2.329-3.679)	<0.001
P for trend		-	<0.001	-	<0.001	-	<0.001
Per SD		1.602 (1.518-1.692)	<0.001	1.524 (1.438-1.616)	<0.001	1.469 (1.375-1.569)	<0.001
**Hypertension**							
Quartile 1	795 (44.99)	1 (Reference)		1 (Reference)		1 (Reference)	
Quartile 2	795 (57.57)	1.315 (1.190-1.453)	<0.001	1.286 (1.161-1.424)	<0.001	1.285 (1.160-1.424)	<0.001
Quartile 3	770 (68.20)	1.738 (1.569-1.926)	<0.001	1.648 (1.480-1.835)	<0.001	1.630 (1.463-1.817)	<0.001
Quartile 4	634 (75.12)	2.132 (1.912-2.378)	<0.001	1.969 (1.752-2.214)	<0.001	1.888 (1.658-2.151)	<0.001
P for trend		-	<0.001	-	<0.001	-	<0.001
Per SD		1.359 (1.307-1.414)	<0.001	1.321 (1.265-1.380)	<0.001	1.312 (1.249-1.378)	<0.001
**Chronic kidney disease**							
Quartile 1	290 (12.89)	1 (Reference)		1 (Reference)		1 (Reference)	
Quartile 2	511 (23.52)	1.195 (1.033-1.382)	0.016	1.065 (0.918-1.236)	0.400	1.065 (0.917-1.236)	0.405
Quartile 3	593 (27.31)	1.177 (1.020-1.357)	0.025	0.995 (0.857-1.155)	0.951	0.994 (0.851-1.160)	0.940
Quartile 4	608 (28.99)	1.266 (1.097-1.461)	0.001	1.020 (0.874-1.191)	0.795	1.018 (0.858-1.207)	0.833
P for trend		-	0.005	-	0.839	-	0.860
Per SD		1.078 (1.029-1.131)	0.002	1.015 (0.961-1.071)	0.584	1.021 (0.960-1.085)	0.501

Model 1: adjusted for age and gender

Model 2: Model 1 + current smoking, diabetes (prediabetes for the outcome of diabetes), hypertension (elevated BP for the outcome of hypertension), prevalent CVD, family history of diabetes (for the outcome of diabetes), non-HDL-C, lipid-lowering drug use, pulse rate, and serum creatinine

Model 3: model 2 + metabolic syndrome

*Abbreviations*: *SD* Standard deviation, *CVD* Cardiovascular disease, *BP* Blood pressure, *HDL-C* High-density lipoprotein cholesterol

**Fig. 3:**
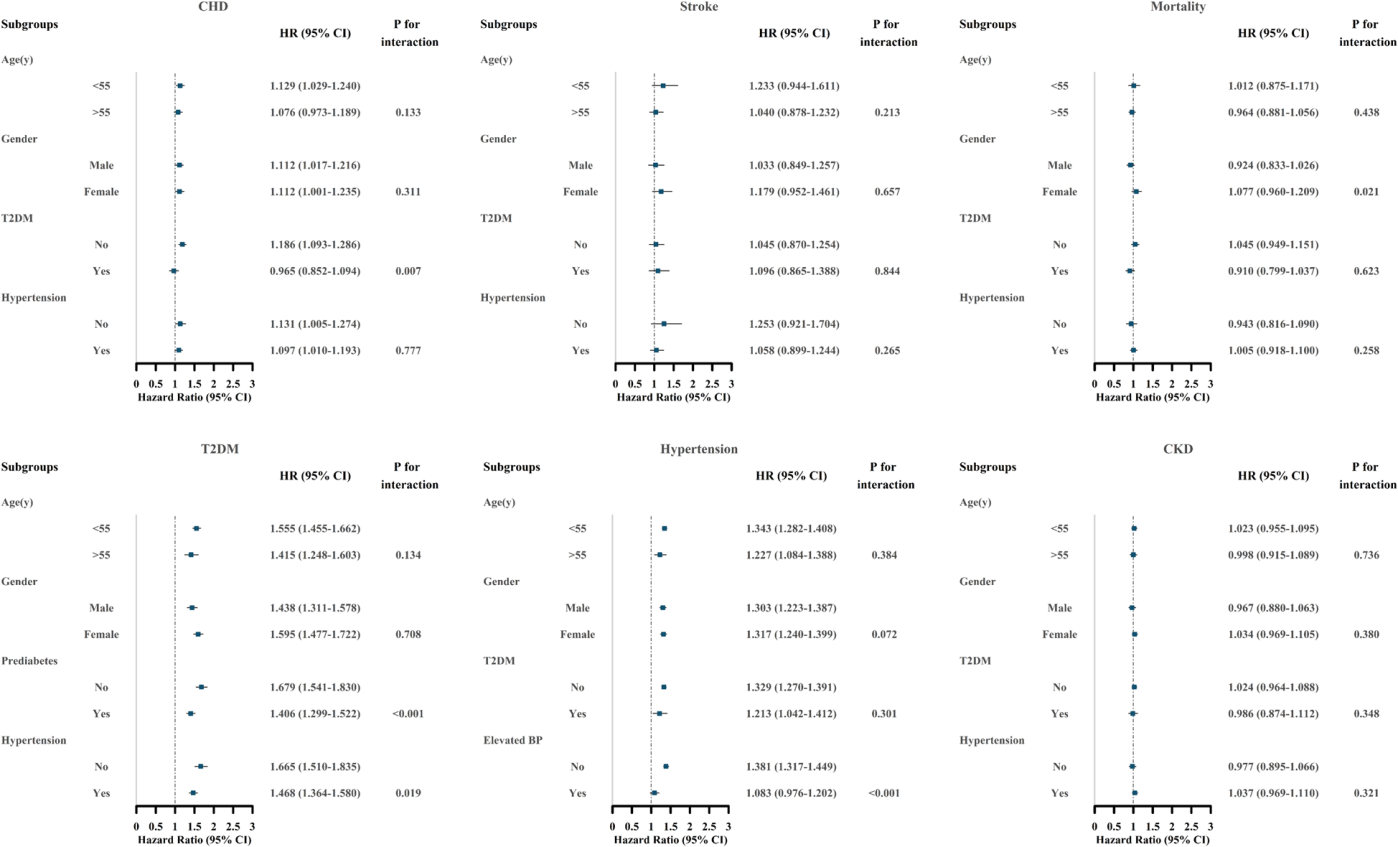
Subgroup analyses for the association of METS-IR per 1-SD increase with the risk of clinical outcomes. Adjusted for age, gender, current smoking, diabetes (prediabetes for the outcome of diabetes), hypertension (elevated BP for the outcome of hypertension), prevalent CVD (family history of CVD for the outcomes of stroke and CHD), family history of diabetes (for the outcome of diabetes), non-HDL-C, lipid-lowering drug use, pulse rate, and serum creatinine. *Abbreviations*: METS-IR, Metabolic Score for Insulin Resistance; CVD, cardiovascular disease; CHD, coronary heart disease; T2DM, type 2 diabetes; CKD, chronic kidney disease; BP, blood pressure; HDL-C, high-density lipoprotein cholesterol; HR, hazard ratio; CI, confidence interval; SD, standard deviation

The association between a 1-SD increase in METS-IR and the risks of CHD, stroke, all-cause mortality, diabetes, hypertension, and CKD was analyzed using stratified analysis based on age, gender, diabetes status (prediabetes for the diabetes outcome), and hypertension status (elevated blood pressure for the hypertension outcome) (Fig. 3). Significant interactions were found between METS-IR and diabetes on CHD risk, METS-IR and gender on mortality risk, METS-IR and prediabetes as well as hypertension on diabetes risk, and METS-IR and elevated BP on hypertension risk (all *P* for interactions < 0.05).

In a secondary analysis, we assessed the association of METS-IR with outcomes in individuals without diabetes and/or METS (Table 4); accordingly, HRs associated with each 1-SD greater METS-IR were 1.178 (1.025–1.354), 1.678 (1.501–1.876), and 1.348 (1.274–1.426) for CHD, diabetes, and hypertension, respectively.

**Table 4:** Hazard ratios and 95% confidence intervals of incident coronary heart disease, stroke, all-cause mortality, diabetes, hypertension, and chronic kidney disease associated with the Metabolic Score for Insulin Resistance in individuals without diabetes and/or metabolic syndrome

Outcomes	Events (%)		
		HR (95% CI) per 1-SD increase	*P* value
CHD	383 (5.79)	1.178 (1.025-1.354)	0.021
Stroke	80 (1.21)	1.056 (0.750-1.487)	0.753
All-cause mortality	365 (5.41)	0.961 (0.815-1.132)	0.637
Diabetes	588 (9.86)	1.678 (1.501-1.876)	<0.001
Hypertension	2358 (55.13)	1.348 (1.274-1.426)	<0.001
CKD	966 (16.82)	1.043 (0.949-1.147)	0.376

Adjusted for age, gender, current smoking, prediabetes, hypertension (elevated BP for the outcome of hypertension), prevalent CVD (family history of CVD for the outcomes of stroke and CHD), family history of diabetes (for the outcome of diabetes), non-HDL-C, lipid-lowering drug use, pulse rate, and serum creatinine

*Abbreviations*: *BP* Blood pressure, *HDL-C* High-density lipoprotein cholesterol, *CVD* Cardiovascular disease, *CHD* Coronary heart disease, *CKD* Chronic kidney disease, *HR* Hazard ratio, *CI* Confidence intervals, *SD* Standard deviation

## Discussion

In this prospective cohort study of an Iranian population over a decade of follow-up, we investigated how METS-IR associates with several clinical outcomes, offering a unique perspective due to our study’s comprehensive dataset and long follow-up period. We assessed how METS-IR is related to the incidence of CHD, stroke, mortality, diabetes, hypertension, and CKD after adjustment for a large set of covariates. We found that an increasing value of METS-IR had a significant association with incident CHD, diabetes, and hypertension. Furthermore, we found a U-shaped association between METS-IR and the risk of all-cause mortality. In those without METS and/or diabetes, the association of METS-IR with CHD, diabetes, and hypertension remained significant.

Similar to our findings, several studies, particularly in East Asian populations, have demonstrated a significant association between METS-IR with incident CVD and its subtypes in the general population. Yoon et al. conducted a prospective cohort study among 17,943 Korean individuals without diabetes to assess the prognostic significance of METS-IR in ischemic heart disease (IHD). They showed that a higher METS-IR was significantly associated with incident IHD and that this index had a better predictive value than METS [34]. A cross-sectional study of individuals without CVD revealed a J-curve correlation between METS-IR and subclinical myocardial infarction [35].

The association of METS-IR with CVD was also evaluated in specific populations; for instance, METS-IR was shown to be associated with approximately 30% increased risk of new-onset CHD and stroke in patients suffering from hypertension and obstructive sleep apnea [36]. Another cohort study among Chinese hypertensive patients, over 4.8 years of follow-up, revealed that METS-IR increased the risk of incident stroke and its ischemic subtype by 80% and 96%, respectively [18]. We extended the previous studies by showing that METS-IR, in a population from the MENA region, was associated with incident CHD among individuals without diabetes and/or METS. Potential mechanisms for the link between METS-IR and CVD may be functional impairment of endothelial cells, lipid abnormalities, and inflammation. Endothelial dysfunction resulting from enhancement in reactive oxygen species and reduced nitric oxide generation would lead to hypertension [37].

We found a U-shaped association between METS-IR and the risk of all-cause mortality. In line with our findings, Wang et al. demonstrated that METS-IR had non-linear and negative associations with all-cause and CVD-associated deaths in patients with diabetes [38]. In contrast, a study on 5,551 individuals without diabetes illustrated that a higher risk of all-cause and CVD-associated death was detected in those with higher HOMA-IR [39]. Interestingly, Kim et al. claimed that obese individuals with high HOMA-IR had a lower risk of all-cause and CVD-associated death, whereas high HOMA-IR was associated with a higher risk of all-cause and CVD-associated death in lean individuals [40]. Li et al. showed a U-shaped association between TyG index and the risk of all-cause mortality among US adults with prevalent CVD, showing that TyG index levels were associated with the lowest risk of all-cause mortality ranging from 8.83 to 9.06 [41].

In our study, the paradoxical association between METS-IR and all-cause mortality may be related to the residual effect of other conditions, such as malnutrition related to poor socioeconomic status, inflammation, and sarcopenia [42, 43], similar to the inverse association that we previously demonstrated regarding triglycerides levels and mortality events [44]. Low FPG is associated with an increased risk of all-cause mortality and CVD in individuals without baseline CVD or diabetes [45]. Hypoglycemia can induce inflammation by enhancing platelet aggregation, activation, and degranulation, along with an increase in vWF and VIII levels, leading to CVD and its adverse events [46, 47]. It has also been demonstrated that lower TG levels are associated with chronic illness [44, 48]. As pancreatic cells are important coordinators in maintaining glucose and lipid homeostasis, we speculate that pancreatic cellular dysfunction may play a part in the positive association observed between low METS-IR and all-cause mortality.

Altogether, there are inconsistent findings concerning the association between IR and mortality, which can be attributed to the heterogeneity of the study population characteristics, presence of chronic illnesses among individuals, different adjustment levels, methodology and design of studies, and different methods of IR assessment.

We found that greater METS-IR, as a categorical or continuous variable, was independently associated with incident diabetes, even among those without METS at baseline. Levels of METS-IR greater than 35.8 were associated with a significantly higher risk of incident diabetes, which was independent of METS. Furthermore, the association between METS-IR and incident diabetes was more pronounced in those without prediabetes or hypertension. Zheng et al. detected an approximately 80% higher risk of incident diabetes for each unit increase in METS-IR in the general population. Moreover, the authors found a significant association between relative and absolute METS-IR change and incident diabetes [49]. However, a 12-year follow-up study claimed that METS-IR cannot predict future prediabetes or diabetes [50]. The mechanism behind this association can be attributed to IR and dysfunction of islet β-cells. As glucose levels rise, islet β-cells generate more reactive oxygen species, contributing to functional impairment of β-cells, which, in turn, leads to diabetes development [51].

We detected a higher risk of incident hypertension in those with higher METS-IR values. Indeed, individuals with METS-IR ≥ 35.8 had a significantly greater risk of incident hypertension, even after further adjustment for METS. Additionally, the association between METS-IR and the risk of hypertension was more prominent in those without elevated BP compared to those with elevated BP. Chavolla et al. found an enhanced risk of incident hypertension for those with METS-IR ≥ 46.4 in 6850 normotensive individuals over 3 years of follow-up. Moreover, METS-IR had higher predictive ability for hypertension compared to other IR indices, including HOMA-IR, TyG, and TG/HDL-C [52]. A meta-analysis of 8 observational East Asian studies revealed that the highest versus lowest category of METS-IR was associated with a 67% greater risk of hypertension and each unit increase in METS-IR was associated with a 15% higher hypertension risk [20].

In the current study, the association of METS-IR with CKD did not remain significant in the fully adjusted model. By far, few studies have investigated the association of METS-IR with CKD. A cross-sectional analysis of 881 Japanese individuals showed that every 10-unit increase in METS-IR was associated with 2.54 units (95% CI: -4.04 to -1.05) decrease in eGFR [53]. A very recent investigation among a total of 9261 Korean adults aged 40–69 years compared the predictive value of HOMA-IR and METS-IR in terms of CKD prevalence as well as its incidence; it was reported that METS-IR had superiority in predicting CKD incidence over HOMA-IR [54].

The findings of this study have significant implications for clinical practice and public health policies in the MENA region. Using the METS-IR as a screening tool, healthcare providers can identify individuals at high risk for adverse cardiometabolic outcomes, enabling timely and targeted interventions. This proactive approach can help design personalized lifestyle modification programs more effectively than generalized recommendations. Furthermore, policymakers can use these insights to allocate resources more efficiently, develop region-specific guidelines for managing insulin resistance, and promote public health initiatives that increase awareness about the importance of metabolic health [10].

## Strengths and limitations

The current study, for the first time in the MENA region, examined the association of METS-IR, a novel and non-insulin-based surrogate of IR, with clinical outcomes including CHD, stroke, all-cause mortality, diabetes, hypertension, and CKD in a large prospective population-based cohort study. On the other hand, some limitations should be acknowledged. First, although we adjusted for several well-known risk factors, the residual confounding may still be present; future research should assess the influence of unmeasured factors like diet, genetics, physical activity, sleep duration, and environmental exposures on the relationship between METS-IR and cardiometabolic outcomes. Also, the relationship between the different trend tracks of METS-IR and health outcomes remains unclear. In future studies, assessing the relationship between METS-IR dynamic trajectories and clinical outcomes would help enhance the validity of the results. Finally, it is not clear whether these findings can be generalized to other ethnicities.

## Conclusions

In conclusion, our study conducted on a population from the MENA region, known for its high burden of cardiometabolic disorders, revealed significant associations between increasing levels of METS-IR, a novel index for measuring insulin resistance, and heightened risks of incident CHD, diabetes, and hypertension. Moreover, we observed a U-shaped relationship between METS-IR levels and the risk of all-cause mortality. Importantly, these associations remained significant even after adjusting for METS.

## Data Availability

The datasets used and/or analyzed in the study are available from the corresponding authors upon reasonable request.

## Abbreviations

NCD: Non-communicable disease
2 h-PG: 2-hour post-challenge plasma glucose
IHD: Ischemic heart disease
METS-IR: Metabolic Score for Insulin Resistance
CHD: Coronary heart disease
CKD: Chronic kidney disease
METS: Metabolic syndrome
IR: Insulin resistance
CVD: Cardiovascular disease
CAC: Coronary artery calcium
TyG: Triglyceride-glucose
HOMA-IR: Homeostatic Model Assessment of Insulin Resistance
WHTR: Waist-to-height ratio
WC: Waist circumference
TLGS: Tehran Lipid and Glucose Study
FPG: Fasting plasma glucose
eGFR: Estimated glomerular filtration rate
OGTT: Oral glucose tolerance test
SBP: Systolic blood pressure
DBP: Diastolic blood pressure
BP: Blood pressure
TG: Triglycerides
HDL-C: High-density lipoprotein cholesterol
SD: Standard deviation
HR: Hazard ratio
CI: Confidence interval
